# Radiologist opinions regarding reporting incidental coronary and cardiac calcification on thoracic CT

**DOI:** 10.1259/bjro.20210057

**Published:** 2022-03-11

**Authors:** Michelle C Williams, Jonathan Weir-McCall, Alastair J Moss, Matthias Schmitt, James Stirrup, Ben Holloway, Deepa Gopalan, Aparna Deshpande, Gareth Morgan Hughes, Bobby Agrawal, Edward Nicol, Giles Roditi, James Shambrook, Russell Bull

**Affiliations:** ^1^ BHF Centre for Cardiovascular Science and Edinburgh Imaging, University of Edinburgh, Edinburgh, UK; ^2^ University of Cambridge School of Clinical Medicine, Cambridge, UK; ^3^ British Heart Foundation Cardiovascular Research Centre, University of Leicester, Leicester, UK; ^4^ North West Heart Centre, Manchester University NHS Foundation Trust, Manchester, UK; ^5^ Royal Berkshire NHS Foundation Trust, Reading, UK; ^6^ Queen Elizabeth Hospital Birmingham, Birmingham, UK; ^7^ Imperial College London, London, UK; ^8^ Glenfield Hospital, University Hospitals of Leicester, Leicester, UK; ^9^ Plymouth Hospitals NHS Trust, Plymouth, UK; ^10^ Royal Papworth Hospital, Cambridge, UK; ^11^ Royal Brompton and Harefield NHS Foundation Trust Departments of Cardiology and Radiology, UK; National Heart and Lung Institute, Faculty of Medicine, Imperial College, London, London, UK; ^12^ Dept. of Radiology, Glasgow Royal Infirmary, NHS Greater Glasgow & Clyde, Glasgow, UK; Institute of Cardiovascular and Medical Sciences, University of Glasgow, Glasgow, UK; ^13^ Southampton General Hospital, Southampton, UK; ^14^ Royal Bournemouth Hospital, Bournemouth, UK

## Abstract

**Objectives:**

Coronary and cardiac calcification are frequent incidental findings on non-gated thoracic computed tomography (CT). However, radiologist opinions and practices regarding the reporting of incidental calcification are poorly understood.

**Methods:**

UK radiologists were invited to complete this online survey, organised by the British Society of Cardiovascular Imaging (BSCI). Questions included anonymous information on subspecialty, level of training and reporting practices for incidental coronary artery, aortic valve, mitral and thoracic aorta calcification.

**Results:**

The survey was completed by 200 respondents: 10% trainees and 90% consultants. Calcification was not reported by 11% for the coronary arteries, 22% for the aortic valve, 35% for the mitral valve and 37% for the thoracic aorta. Those who did not subspecialise in cardiac imaging were less likely to report coronary artery calcification (*p* = 0.005), aortic valve calcification (*p* = 0.001) or mitral valve calcification (*p* = 0.008), but there was no difference in the reporting of thoracic aorta calcification. Those who did not subspecialise in cardiac imaging were also less likely to provide management recommendations for coronary artery calcification (*p* < 0.001) or recommend echocardiography for aortic valve calcification (*p* < 0.001), but there was no difference for mitral valve or thoracic aorta recommendations.

**Conclusion:**

Incidental coronary artery, valvular and aorta calcification are frequently not reported on thoracic CT and there are differences in reporting practices based on subspeciality.

**Advances in knowledge:**

On routine thoracic CT, 11% of radiologists do not report coronary artery calcification. Radiologist reporting practices vary depending on subspeciality but not level of training.

## Introduction

**Figure 1.**
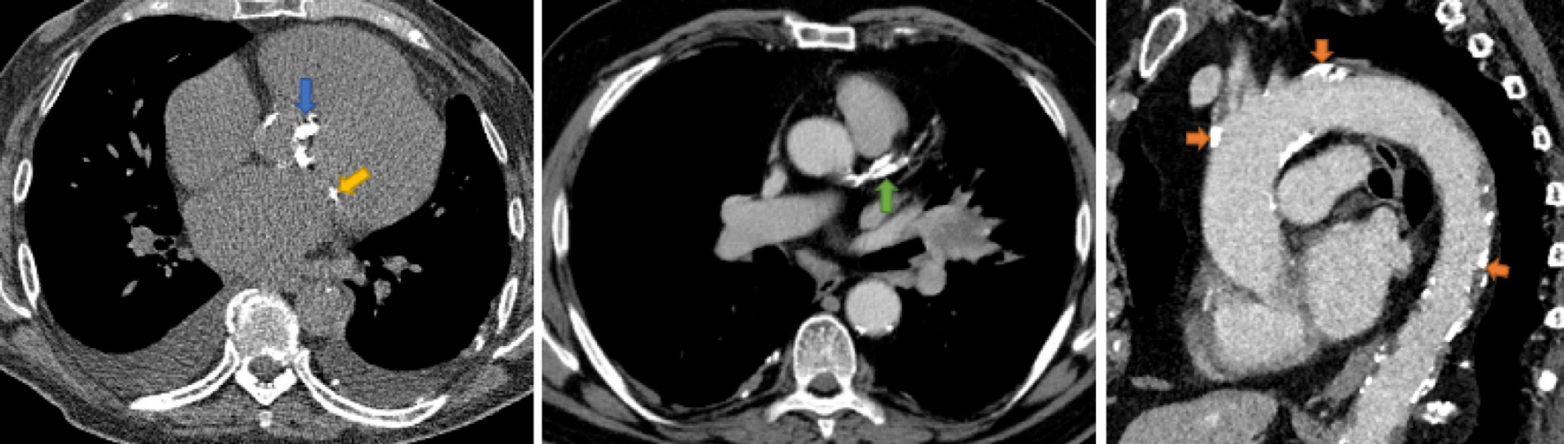
Examples of aortic valve calcification (blue arrow), mitral valve calcification (yellow arrow), coronary artery (green arrow) and thoracic aorta calcification (orange arrow).

Calcification in the coronary arteries, cardiac valves and thoracic aorta are frequent incidental findings (Figure 1) on routine non-gated thoracic computed tomography (CT)^1–11^ and may indicate important underlying pathologies. However, little is known about the reporting practices of radiologists regarding these incidental findings.

Cardiac and vascular calcification share overlapping risk factors and pathogenesis. Coronary artery calcification is a marker of atherosclerosis and its presence and extent are associated with increased cardiovascular and all-cause mortality.^12–21^ Similarly, aortic valve calcification is a marker of aortic sclerosis or stenosis and its severity is associated with the degree of aortic valve disfunction.^22–24^ The clinical significance of mitral calcification is less certain, as although mitral valve calcification is associated with mitral valve dysfunction mitral annular calcification is a frequent finding that is rarely associated with mitral valve dysfunction. Calcification in the thoracic aorta is also a frequent finding in asymptomatic patients, and its severity may be associated with the severity of coronary artery disease and cardiovascular mortality.^11,25^ Although the reporting of coronary artery calcification on non-gated thoracic CT is supported by national and international guidelines, it is frequently not reported.^1–3,26^

This survey aims to investigate the current practices and opinions of UK radiologists regarding the reporting of cardiac and vascular calcification on routine non-gated thoracic CT.

## Methods

### Survey design

This survey was organised by the British Society of Cardiovascular Imaging/British Society of Cardiac Computed Tomography (BSCI/BSCCT). The executive committee of the BSCI/BSCCT acted as the steering committee for the survey. Survey questions were designed and refined by the BSCI/BSCCT executive committee. An electronic platform (Survey Monkey) was used to collect anonymous survey data. When more than one option was provided as an answer to a question, these were provided in a random order. Invitations to participate were sent to all BSCI/BSCCT members and they were asked to forward the survey to all radiologists working in their hospitals.

### Survey questions

Survey questions included information about the respondent’s subspecialty and level of training. For each of coronary artery calcification, aortic valve calcification, mitral calcification (valve and/or annulus), and thoracic aorta calcification they were asked whether they routinely report its presence, what factors affected whether they would report its presence and if management recommendations were provided. For coronary artery calcification, they were asked what method they use to report it (visual assessment on a per patient level, visual assessment on a per vessel level, semi-quantitative score, or Agatston score). For aortic valve and mitral calcification, they were asked whether they would recommend echocardiography. Respondents were free to choose not to answer certain questions. Additional comments were solicited at the end of the survey.

### Statistical analysis

Statistical analysis was performed using R v. 4.0.1 (R Foundation for Statistical Computing, Vienna, Austria). Survey responses which only included information on subspecialty and job description questions were deemed incomplete and excluded from the analysis. Number and percentage are presented for categorical data with statistical significance assessed using a Pearson’s chi-square test. Binomial logistic regression analysis was performed to assess the impact of level of training and subspecialisation on reporting practices. A statistically significant difference was defined as a two-sided *p*-value < 0.05.

## Results

### Demographic information

After excluding incomplete survey responses (*n* = 12), there were 200 survey responses that were included in this analysis. This included 20 (10%) trainees and 180 (90%) consultants. Of the consultants, 25% (*n* = 45) were less than 5-year post-training, 23% (*n* = 41) were 5 to 10 years post-training, and 52% (*n* = 94) were more than 10 years post-training. Subspecialty practice/training was reported by 50% (*n* = 100) for cardiac imaging, 57% (*n* = 114) for thoracic imaging, and 16% (*n* = 33) for vascular imaging. 30% (*n* = 59) of respondents did not subspecialise in cardiac, thoracic, or vascular imaging.

### Coronary artery calcification

**Figure 2.**
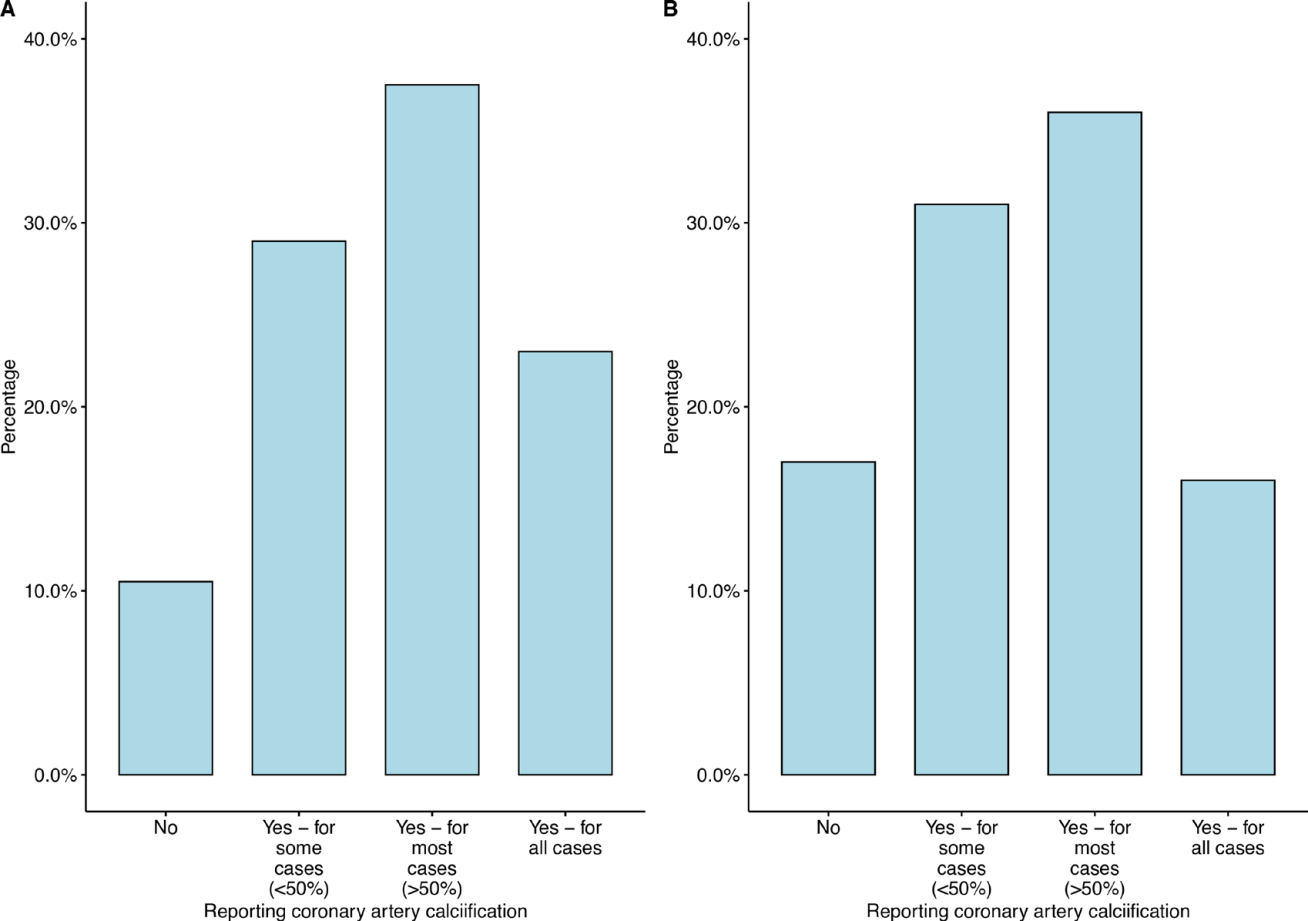
Reporting of coronary artery calcification by (**A**) all survey respondents and (**B**) those who did not subspecialise in cardiac imaging.

Coronary artery calcification was reported for all cases by 23% of radiologists, for most cases by 38% of radiologists, and some cases by 29% of radiologists (Table 1). Coronary artery calcification was not reported by 11% of all survey respondents (Figure 2), and those who were not subspecialised in cardiac imaging were less likely to report coronary artery calcification on routine thoracic CT (17% of non-cardiac specialists did not report coronary artery calcification, *n* = 17/100 *vs* 4% of cardiac specialists did not report coronary artery calcification, *n* = 4/100; *p* = 0.005). Trainees were more likely to report all, most or some coronary artery calcification (95%, *n* = 19/20), compared to consultants with less than 5 years of experience (93%, *n* = 42/45), consultants with 5 to 10 years of experience (93%, *n* = 38/41) and consultants with more than 10 years of experience (85%, *n* = 80/94, Table 2), but these differences did not reach statistical significance (*p* = 0.293).

**Table 1.** Frequency of reporting of cardiac and vascular calcification

	Coronary artery calcification	Aortic valve calcification	Mitral valve calcification	Thoracic aorta calcification
Number	200	198	196	195
Yes - for all cases	46 (23%)	47 (24%)	33 (17%)	12 (6%)
Yes - for most cases (>50%)	75 (38%)	45 (23%)	-	24 (12%)
Yes - for some cases (<50%)	58 (30%)	63 (32%)	93 (47%)	85 (43%)
No	21 (11%)	43 (22%)	70 (35%)	74 (37%)

**Table 2.** Impact of level of training on reporting of cardiac and vascular calcification

	Trainee	Consultant		
		<5 years	5–10 years	>10 years
Coronary artery calcification ^a^	19 (95%)	42 (93%)	39 (93%)	80 (85%)
Aortic valve calcification ^a^	15 (75%)	34 (76%)	34 (83%)	72 (77%)
Mitral valve calcification ^a^	12 (60%)	29 (64%)	23 (56%)	62 (66%)
Thoracic aorta calcification ^a^	12 (65%)	28 (62%)	29 (71%)	51 (54%)

a Combined “Yes - for all cases”, “Yes – for most cases” and “Yes – for some cases”

**Figure 3.**
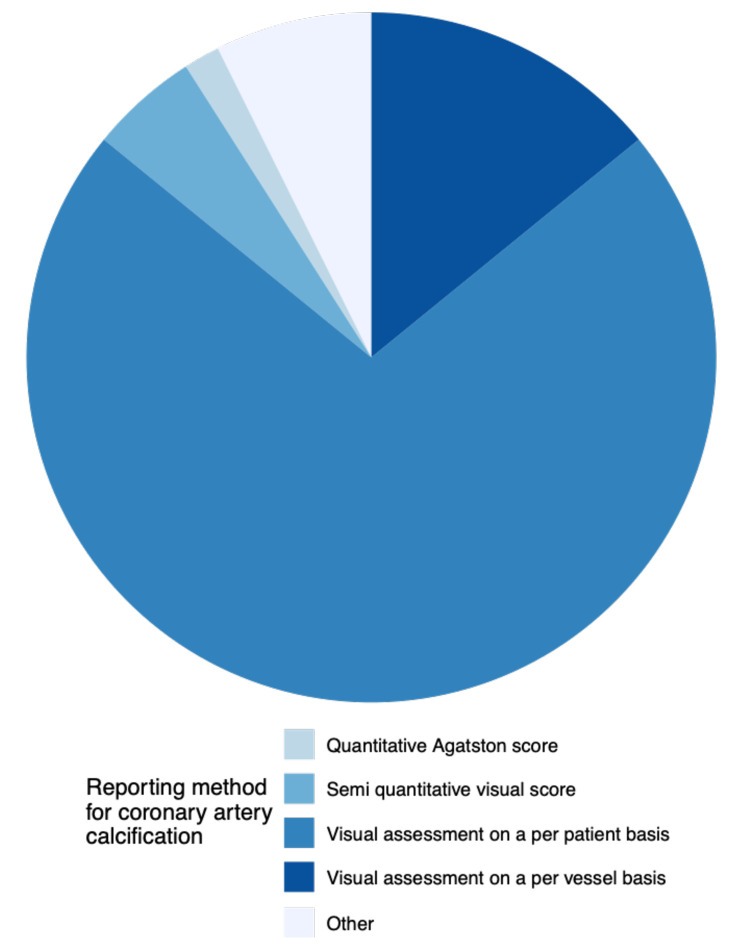
Reporting methods used by respondents who report coronary artery calcification.

**Figure 4.**
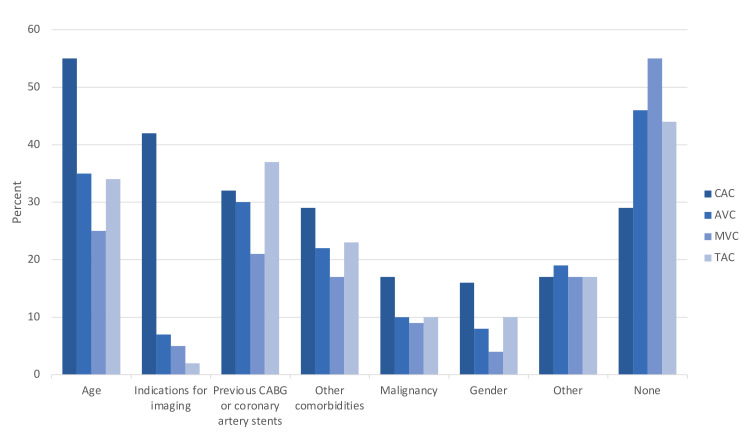
Factors that influence reporting of coronary artery calcification (CAC), aortic valve calcification (AVC), mitral valve calcification (MVC) and thoracic aorta calcification (TAC).

Visual assessment on a per patient basis was the most frequent method used to report coronary artery calcification (Figure 3). Age and indication for imaging were the most frequent factors to influence whether coronary artery calcification was reported, influencing 55 and 42% of respondents, respectively (Figure 4).

Management recommendations for coronary artery calcification findings were provided for all cases by 7% (*n* = 14), for most cases 8% (*n* = 15) and for some cases by 22% (*n* = 45). Management recommendations were not provided by 63% (*n* = 126) and were less likely to be provided by those who did not subspecialise in cardiac imaging (79%, *p* < 0.001). Free text comments highlighted concerns regarding the potential clinical benefit of reporting these findings, or the potential to increase unnecessary further investigations.

### Aortic valve calcification

Aortic valve calcification was reported for all cases by 24% of radiologists, for most cases by 23% of radiologists, and some cases by 1% of radiologists (Table 1). Aortic valve calcification was not reported by 22% of all survey respondents, and those who did not subspecialise in cardiac imaging were less likely to report aortic valve calcification (31% of non-cardiac specialists did not report coronary artery calcification, *n* = 31/100 *vs* 12% of cardiac specialists did not report coronary artery calcification, *n* = 12/100; *p* = 0.001). Level of training did not impact reporting of aortic valve calcification (Table 2, *p* = 0.293). Age and previous cardiac intervention were the most frequent factors influencing reporting of aortic valve calcification, influencing 35 and 30% of respondents, respectively. Echocardiogram was recommended following the identification of aortic valve calcification by 4% (*n* = 8) for all cases, 12% (*n* = 24) for most cases and 20% (*n* = 40) for some cases. Recommendations for echocardiography were not provided by 63% (*n* = 126) and were less likely to be provided by those who did not subspecialise in cardiac imaging (77% of non-cardiac specialists did not provide recommendations for echocardiography, *n* = 77/100 *vs* 49% of cardiac specialists did not provide recommendations for echocardiography, *n* = 49/100; *p* < 0.001)

### Mitral calcification

Mitral calcification (valve and/or annulus) was reported for all cases by 17% of radiologists and for some cases by 47% or radiologists (Table 1). Mitral calcification was not reported by 35% of all survey respondents, and those who did not subspecialise in cardiac imaging were less likely to report mitral calcification (41% of non-cardiac specialists did not report mitral calcification, *n* = 41/100 *vs* 29% of cardiac specialists did not report mitral calcification, *n* = 29/100; *p* = 0.0996), but this difference did not reach statistical significance. Level of training did not impact reporting of mitral calcification (*p* = 0.599, Table 2). Age and previous cardiac intervention were the most frequent factors influencing reporting of mitral calcification, influencing 25 and 21% of respondents, respectively. Echocardiogram was recommended following the identification of mitral calcification by 2% (*n* = 4) for all cases, 3% (*n* = 5) for most cases and 14% (*n* = 28) for some cases. Recommendations for echocardiography were not provided by 80% (*n* = 159) and were less likely to be provided by those who did not subspecialise in cardiac imaging, but this difference did not reach statistical significance (84% of non-cardiac specialists did not provide recommendations for echocardiography, *n* = 84/100 *vs* 75% of cardiac specialists did not provide recommendations for echocardiography, *n* = 77/100; *p* = 0.092).

### Thoracic aorta

Thoracic aortic calcification was reported for all cases by 6% of radiologists, for most cases by 12% of radiologists, and for some cases by 43% of radiologists (Table 1). Thoracic aorta calcification was not reported by 37% of all survey respondents, this was similar for those who did not subspecialise in cardiac imaging and those who did (41% of non-cardiac specialists did not report thoracic aorta calcification, *n* = 41/100 *vs* 33% of cardiac specialists did not report thoracic aorta calcification, *n* = 33/100; *p* = 0.420). Level of training did not impact reporting of thoracic aortic calcification (*p* = 0.491, Table 2). Age, previous cardiac intervention, and presence of comorbidities were the most frequent factors influencing reporting of thoracic aortic calcification, influencing 34%, 37 and 23% of respondents respectively (Figure 3).

### Free text comments

Free text comments were provided by 33 (17%) respondents. Of these, 7 (21%) were regarding the importance of the topic of this survey. Two of the comments (6%) were concerned about the potential for reporting incidental calcification to cause patient anxiety. While 5 (15%) comments were concerned that reporting incidental calcification would cause unnecessary investigations and increased workload, 2 (6%) thought that reporting incidental calcification would be ignored by referrers. One (3%) comment was concerned about potential medicolegal implications. Seven (21%) of the comments suggested that clear guidelines would be useful for their clinical practice, including reporting templates and clear pathways for referrers. The remaining comments provided further detail on their current reporting practices.

## Discussion

This survey found that incidental calcification is frequently not reported on routine thoracic CT and that subspecialisation, but not level of training, influenced reporting practices. Subspecialisation in cardiac imaging influenced reporting of incidental calcification in the coronary arteries, aortic valve, and mitral valve, but not in the thoracic aorta. The primary factors which influenced reporting of calcification were age and indication for imaging. Management recommendations were provided infrequently and were less likely to be provided by those who did not subspecialise in cardiac imaging.

Extensive research has shown that coronary artery calcification is an effective marker of the presence of coronary artery disease and is associated with both cardiovascular and all-cause mortality in patients undergoing dedicated cardiac imaging or routine thoracic imaging.^4,12–21,27–31^ Nevertheless, our survey showed that coronary artery calcification was not reported by 11% of survey respondents. At present, there remains debate in the radiology community regarding the reporting of cardiac calcification on routine thoracic CT. In our survey comments included concerns around patient anxiety, unnecessary investigations and increased workload for radiology and cardiology departments. Other concerns that have been raised include cost-effectiveness, ethical and medicolegal implications, additional reporting time and unknown clinical utility. Randomised controlled trials of management changes based on incidental calcification have not yet been completed. The Risk Or Benefit IN Screening for Cardiovascular diseases (ROBINSCA) randomised controlled trial is currently assessing the clinical impact of screening asymptomatic individuals with coronary artery calcium scoring compared to either screening based on traditional cardiovascular risk factors or no screening.^32^ Information on cardiovascular outcomes in this trial are awaited, but early publications show that basing medication choices on calcium scoring reduced the number of patients receiving preventative medication compared to screening using traditional cardiovascular risk factors.^32^ The results of this and other ongoing research will shape future guidelines in this area.

The recently published BSCI/BSCCT consensus statement on reporting incidental coronary, aortic valve and cardiac calcification on non-gated thoracic CT aims to address some of the concerns raised in this survey.^33^ For coronary artery calcification, it suggests a simple patient-based score for coronary artery calcification (none, mild, moderate, severe) and highlights that many patients with incidental calcification do not require further investigation.^33^ Repeating this survey after the publication of this consensus statement will be important to assess future changes in the opinions of radiologists throughout the UK. The Society of Cardiovascular Computed tomography have also published international guidelines which recommend that coronary artery calcification should be reported on all non-gated thoracic CT.^34^ There are currently no reporting guidelines that address the issue of thoracic aortic calcification on non-gated thoracic CT, highlighting on-going uncertainty in this area.

We found that on thoracic CT radiologists were less likely to report non-coronary cardiovascular calcification compared to coronary calcification. The degree of aortic valve calcification on electrocardiogram-gated CT is associated with the severity of aortic valve disease and reporting this is part of international guidelines for the management of valvular heart disease.^35^ However, for mitral calcification and thoracic aortic calcification their impact is more debated. Although mitral valve leaflet calcification will indicate valvular dysfunction, mitral annular calcification is more frequently an asymptomatic incidental finding (although in some severe cases is associated with significant valvular dysfunction). The severity of thoracic aortic calcification correlates with coronary artery calcium scores, but it is not independent of coronary artery calcification in the assessment of cardiac events.^36–38^ Further research in these areas will help guide reporting of these incidental findings.

Limitations of this survey include the number of participants, the UK only participants and the unequal proportion of trainees and consultants. Those who completed this survey are more likely to be interested in this topic (selection bias), and therefore the true frequency of reporting is likely to be lower than that presented by this survey. As this was a survey of reporting practices rather than directly assessing CT reports there is the potential for recall bias to impact our results. We did not ask participants whether they reported findings differently on non-contrast compared to contrast enhanced scans and we did not discriminate between mitral valve and mitral annular calcification. We also did not enquire whether respondents worked in district general, tertiary care or academic departments, which may also influence reporting practices. Participants were also free not to answer all of the questions in the survey, meaning some of the data is incomplete. The survey was extensively reviewed by members of the BSCI/BSCCT executive committee, which includes consultant and trainee radiologists and cardiologists, but as this was not an externally validated survey the way that the questions were asked could have influenced the responses. Nevertheless, this survey provides an interesting insight into the practices of radiologists regarding the reporting of incidental calcification.

In conclusion, this survey found that coronary, valvular and aortic calcification is frequently not reported by UK radiologists. Subspecialisation but not level of training impacted reporting practices.
